# Caregiver Wellness Questionnaire: Initial Psychometric Evaluation of a Multidimensional Measure of Well-Being Among a Mixed U.S. Caregiver Sample

**DOI:** 10.3390/healthcare14193247

**Published:** 2026-10-01

**Authors:** Eboni I. Green, Kristin Wiginton

**Affiliations:** Healthcare Administration, College of Management and Human Potential, Walden University, Minneapolis, MN 55401, USA; kristin.wiginton@mail.waldenu.edu

**Keywords:** caregiver wellness, caregiver, caregiver support, caregiver wellness questionnaire, holistic caregiver wellness, caregiver wellness: U-model, caregiver wellness theory, integrated wellness, empowerment and caregiving, resilience and caregiving

## Abstract

**Highlights:**

**What are the main findings?**

- Significant correlations among wellness domains support the theoretical premise that caregiver wellness is multidimensional and that domains influence one another.

**What are the implications of the main findings?**

- The Caregiver Wellness Questionnaire provides a psychometrically supported instrument for measuring its multidimensional domains.

**Abstract:**

**Background/Objectives**: The Caregiver Wellness: U-Model Questionnaire offers a self-guided approach to assessing caregiver well-being and provides resources designed to empower caregivers to embrace self-care. The instrument consists of 27 items organized into nine wellness dimensions: occupational wellness, physical wellness, psychological wellness, intellectual wellness, social wellness, resilience, empowerment, spiritual wellness, and financial wellness. The purpose of this study was to evaluate the validity and reliability of the Caregiver Wellness: U-Model Questionnaire for measuring the perceived well-being of caregivers. **Methods**: This secondary analysis used a convenience sample of 225 participants who completed the questionnaire between 2022 and 2025; family caregivers and frontline direct care workers could not be distinguished within the dataset. Analyses of the CWQ included internal reliability consistency, exploratory factor analysis (EFA) and subscale correlations for model and subscale refinement. **Results**: McDonald’s omega (0.828) indicated high reliability of the CWQ. The EFA revealed six factors retained on Eigenvalues > 1 and visual inspection of a scree plot. Subscale correlations showed evidence of convergent validity. The original nine subscale questionnaire items were mapped to the corresponding six factors from the structure matrix. The resulting six subscales are Connected Well-Being, Personal Agency, Empowered Adaptive Capacity, Functional Capacity, Resilient Self-Regulation, and Occupational Wellness. **Conclusions**: The findings of this study and the revised six-factor Caregiver Wellness model demonstrate an improved understanding of caregiver wellness as an interplay of relational connectedness, personal empowerment, adaptive resilience, functional resources, self-regulatory processes, and occupational fulfillment.

## 1. Introduction

The ongoing demands of family caregiving can adversely impact caregiver well-being, contributing to emotional strain, psychological distress, and burnout [1,2,3]. Wellness-oriented interventions may help caregivers and helping professionals combat emotional exhaustion and compassion fatigue by strengthening resilience, mobilizing social support, and developing sustainable self-care practices [4,5]. These challenges underscore the need for comprehensive support measures and effective assessment tools that capture caregivers’ wellness needs and can inform appropriate support. The purpose of this study is therefore to evaluate the validity and reliability of the Caregiver Wellness: U-Model Questionnaire (CWQ) for measuring the perceived well-being of family and frontline direct caregivers within the United States.

Caregiver wellness is a multidimensional construct encompassing psychological, physical, social, spiritual, intellectual, occupational, and financial well-being, as well as capacities such as empowerment and resilience. Existing holistic wellness models, designed for the general population, provide a foundation for understanding well-being but have limitations when applied specifically to caregivers. Myers’s Wheel of Wellness serves as a general counseling model [6] and conceptualizes wellness holistically, encompassing multiple dimensions of life and the broader contexts in which individuals function, such as family, community, religion, education, government, media, and global events [4,5]. A widely used self-assessment tool supported by extensive research, it is occasionally applied to family caregivers, even though it was not designed for caregiving populations and does not explicitly address their specific experiences such as burden, role strain, caregiving competence, or resilience.

Likewise, Gelardin’s Career and Family Caregiver Counseling model was developed for holistic health and longevity and has been used in training and certificate programs [7]. The model recognizes that caregiver wellness extends beyond physical health to include emotional, social, spiritual, intellectual, and occupational dimensions but serves largely as a conceptual framework and does not include validated or standardized tools. Neither model was developed with the explicit aim of empowering caregivers to take proactive steps in caring for themselves or building resilience through new approaches to self-care.

To address the identified gap, Green, the first author of this study, developed the Caregiver Wellness: U-Model (CWU), a new framework to address caregiver-specific wellness needs. The CWU adapts wellness theory to the experiences and demands of caregiving. Rather than focusing on caregiver deficits, it acknowledges caregiver strengths, helping them to build upon these and pursue optimal wellness [8]. The CWU conceptualizes wellness as a continuum rather than a fixed endpoint and encourages caregivers to see wellness as an ongoing journey. It acknowledges the unique tasks associated with caregiving and recognizes caregiving as both a personal and collective social responsibility.

This model builds on the research behind the Myers Wheel of Wellness [6] and the foundational principles of the Wellness Behavior and Interaction Model [9], wherein wellness is more a multidimensional construct and not simply the absence of disease [10]. Consistent with this perspective, the CWU incorporates psychological (rather than emotional), physical, social, financial, occupational, intellectual, and spiritual dimensions of wellness. As in traditional wellness theory, these dimensions are interconnected, with no single dimension operating independently. The CWU additionally introduces two critical constructs: *empowerment*, which encourages caregivers to recognize their abilities and take proactive steps toward improving their own wellness, and *resilience*, which builds their strength to withstand challenges and continue providing care effectively [8]. By applying these concepts to the distinct challenges and responsibilities associated with caregiving, the framework offers a caregiver-centered approach to promoting well-being and supporting caregivers in effectively addressing their own health and wellness needs.

The CWU was first conceptualized through a 2009 doctoral dissertation examining the multidimensional health risks and wellness needs of caregivers [11], with findings subsequently disseminated through scholarly and practitioner-focused publications and presentations, including work with the Alzheimer’s Foundation of America (AFA). In 2010, the Caregiver Wellness Domain Compendium (CWDC) was developed to integrate caregiver perspectives, empirical evidence, assessment measures, and evidence-informed strategies, providing the basis for translating the model’s wellness constructs into measurable CWQ items. Initially comprising one item for each of nine caregiver wellness constructs, the questionnaire was progressively refined through feedback from AFA social workers and caregivers and review by family caregivers, frontline direct care workers, and social service professionals [12]. The CWQ subsequently developed into a 27-item self-reflection instrument and was implemented through online caregiver resources such as the Caregiver Support Services website [13].

The current CWU is a conceptual framework in which each construct links to the CWQ’s wellness domains and evidence-informed items. The CWQ is therefore a comprehensive, strength-based tool for caregivers to evaluate their well-being, identify unmet needs, guide resource navigation, and inform the development of supportive interventions. The tool can be used by all caregivers, irrespective of the care recipient’s diagnosis, with a focus on caregivers themselves and on translating self-care into actionable steps. Hence, this study is an initial exploratory psychometric evaluation of the CWQ.

## 2. Materials and Methods

The primary aim of this quantitative study, based on secondary data analysis, was to establish the reliability and validity of the CWQ. Key objectives included evaluating the instrument’s reliability by assessing the consistency of participant responses. The study employed a quantitative, reliability-focused design to examine the internal coherence and stability of responses across questionnaire items. This research was conducted in accordance with the ethical principles outlined in the Declaration of Helsinki and was approved by the Walden University Institutional Review Board.

### 2.1. Setting & Sample

Data for this study were obtained through a secondary analysis of records provided by Caregiver Support Services (CSS), a nonprofit organization that offers evidence-based training, practical tools, and a supportive community to help caregivers provide high-quality care without compromising their own health and well-being. The study utilized a convenience sample of caregivers who completed the online CWQ between 2022 and 2025 (*N* = 225). Participants included both family caregivers and frontline direct care workers; however, the dataset did not distinguish between these groups. The data were originally collected by CSS as part of routine program operations and service delivery rather than for research purposes. Caregivers were invited to complete the questionnaire to identify personal strengths and one key area for improving their well-being. These administrative and programmatic records were subsequently maintained within the organization’s secure database system and later made available for secondary analysis.

Participant consent was waived because this study involved a secondary analysis of an existing de-identified dataset obtained from CSS, a nonprofit organization. The dataset was originally collected for programmatic and service-related purposes and was provided to the researchers without any direct or indirect personal identifiers. Researchers had no contact with participants and did not recruit or interact with individuals whose data were included in the dataset. Because the analysis involved only previously collected, de-identified information and posed no more than minimal risk to participants, obtaining informed consent was not required. The use of secondary, non-identifiable data protected participant privacy and confidentiality throughout the study.

### 2.2. Caregiver Wellness Questionnaire

The CWQ was the instrument for assessing caregiver wellness across nine domains. The CWQ is a multidimensional self-report measure designed to evaluate perceptions of wellness among caregivers and to identify areas of strength and areas requiring improvement. The instrument consists of 27 items organized into nine wellness dimensions, with three items assigned to each dimension. The nine dimensions assessed by the instrument include: occupational wellness, physical wellness, psychological wellness, intellectual wellness, social wellness, resilience, empowerment, spiritual wellness, and financial wellness.

The CWQ was developed based on a holistic understanding of caregiver well-being, recognizing that caregiver wellness extends beyond physical health and encompasses psychological, social, spiritual, occupational, and financial aspects of functioning. Each dimension is intended to provide a focused assessment of a specific component of caregiver wellness while collectively contributing to an overall profile of wellness status.

### 2.3. Item Structure and Response Format

Participants responded to each of the 27 items using a 3-point Likert-type scale consisting of the following response options: 1 = agree, 2 = somewhat agree, and 3 = disagree.

Items were written to reflect experiences, perceptions, or behaviors associated with caregiver wellness. The questionnaire included both positive and negatively worded statements to reduce response bias and encourage thoughtful responses. Negatively worded items were reverse scored prior to analysis to ensure that all items were oriented in the same direction. For negatively worded items, response values were recoded as follows: agree = 3, somewhat agree = 2, and disagree = 1.

This reverse-scoring procedure ensured consistency across all items, such that higher scores uniformly indicated greater wellness concerns or areas for improvement, whereas lower scores reflected stronger wellness functioning.

### 2.4. Subscale Scoring Procedures

The CWQ is scored at the subscale level, with each wellness dimension analyzed independently. Scores for each subscale were calculated by summing responses across the three items associated with that dimension (Table 1). Because each subscale contains three items scored on a 1 to 3 scale, possible subscale scores range from: minimum score of 3 to a maximum score of 9.

Composite scores (lower) closer to 3 indicate a wellness strength and reflect more favorable outcomes within that domain. A composite score (higher) closer to 9 indicates a wellness challenge or area for improvement and suggests the potential need for intervention, support, or additional resources in that domain.

As a result, each of the nine subscales generates an independent wellness profile that can be used to identify specific strengths and opportunities for growth. The instrument is designed to support individualized assessment rather than relying solely on a total composite score.

### 2.5. Procedures

The dataset provided for this study included responses to the CWQ, which served as the primary measure of caregiver wellness. Prior to transferring to the researchers, CSS prepared a fully de-identified dataset to protect participant privacy and confidentiality. All direct identifiers, including participant names, email addresses, and home state, were removed. CSS also reviewed the dataset to eliminate indirect identifiers and combinations of variables that could reasonably be used to identify individual participants. The researchers did not have access to identifiable information at any stage of the study and received only the variables necessary to address the research questions.

The initial version of the Caregiver Wellness Questionnaire (CWQ) consisted of 27 items. During data preparation, one item was identified as containing errors and was removed prior to psychometric testing, resulting in a final set of 26 items for analysis. Following receipt of the dataset, the researchers reviewed the data for completeness, accuracy, missing values, and response distributions. Responses to negatively worded CWQ items were reverse scored according to the instrument scoring protocol, and composite scores were calculated for each of the nine CWQ wellness dimensions. Descriptive analyses were conducted to characterize the sample and summarize item- and subscale-level performance, including means, standard deviations, score ranges, missing data patterns, and floor and ceiling effects.

The resulting de-identified dataset was stored on a password-protected computer accessible only to the researchers and analyzed using appropriate statistical procedures to address the study objectives. The use of an existing, de-identified dataset minimized risks to participants while enabling the examination of caregiver wellness patterns within the CSS caregiver population.

Statistical analyses were conducted to evaluate the psychometric properties of the CWQ, including descriptive characteristics, internal consistency reliability, factor structure, construct validity, and convergent relationships among subscales and items.

### 2.6. Descriptive Statistics and Data Screening

Descriptive statistics were calculated for all CWQ items and subscale scores. Means, standard deviations, score ranges, frequencies, and percentages were examined to assess the distribution of responses and identify potential data quality issues. Missing data patterns, floor effects, and ceiling effects were also evaluated to determine whether the data met assumptions for subsequent analyses.

### 2.7. Internal Consistency Reliability

Internal consistency reliability of the CWQ was evaluated using McDonald’s omega (ω) coefficient for each of the nine wellness subscales and for the overall instrument. McDonald’s omega is recommended as a more robust estimate of internal consistency than Cronbach’s alpha because it does not assume equal factor loadings across items and often provides a more accurate estimate of reliability for multidimensional psychological measures [14]. Omega coefficients of 0.70 or higher were considered indicative of acceptable internal consistency for research purposes, with higher values reflecting stronger reliability evidence.

### 2.8. Factor Structure Assessment and Construct Validity

Construct validity was assessed to determine whether the CWQ measured the underlying dimensions of caregiver wellness as conceptualized in the CWU. Factor-analytic procedures were used to evaluate the questionnaire’s dimensional structure and to examine the extent to which individual items clustered into theoretically meaningful wellness domains.

Factor loadings, cross-loadings, item communalities, and overall factor interpretability were examined to assess the appropriateness of item placement within factors. The resulting factor structure was reviewed for conceptual coherence and alignment with the theoretical framework underlying the questionnaire.

The factor-analytic process provided evidence regarding the extent to which the CWQ reflected the multidimensional nature of caregiver wellness and supported evaluation of the instrument’s construct validity. Alignment between the identified factor structure and the conceptual framework was considered an important indicator that the questionnaire measured the intended wellness domains.

Exploratory Factor Analysis (EFA) was selected to assess the underlying dimensional structure of the CWQ. Although the instrument was developed based on the CWU and its nine theoretically defined wellness dimensions, the factor structure of the questionnaire had not been previously established through psychometric testing. EFA is recommended during the initial stages of instrument validation because it allows researchers to examine how items group together based on observed response patterns without imposing a predetermined structure on the data [15,16]. This approach helps identify latent constructs, evaluate item-factor relationships, detect cross-loading items, and determine whether the emerging factor structure is conceptually consistent with the intended domains of caregiver wellness. By allowing the data to reveal underlying patterns, EFA provides foundational evidence of construct validity and informs subsequent refinement of the instrument and testing of its factor structure in future confirmatory analyses.

EFA was particularly appropriate because the study represented the first large-scale psychometric evaluation of the CWQ. Although the questionnaire was theory-driven, empirical examination of the factor structure was necessary to determine whether respondents interpreted and organized the items in a manner consistent with the proposed wellness domains. Therefore, EFA served as an essential step in evaluating construct validity and guiding instrument refinement.

### 2.9. Correlation Analysis

Pearson correlation coefficients were calculated to examine relationships among CWQ subscales and between individual items and their assigned subscales. Item-to-subscale correlations were reviewed to determine whether items demonstrated stronger associations with their intended wellness dimension than with unrelated dimensions. Subscale intercorrelations were also evaluated to assess the extent to which wellness dimensions were related while remaining conceptually distinct constructs.

### 2.10. Microsoft Copilot

Researchers used Microsoft Copilot (Microsoft 365 Copilot; Microsoft Corporation, Redmond, WA, United States) [17] to generate potential titles for the constructs.

## 3. Results

### 3.1. Descriptive Statistics and Data Screening

Responses showed considerable variability across wellness domains, with mean scores for each question ranging from 1.47 to 2.33 on a 1–3 scale, suggesting that caregivers experienced differing levels of wellness depending on the specific dimension assessed (Table 2). The highest mean scores were observed in questions focusing on Social Well-being (A2, M = 2.33), Intellectual Wellness (A2, M = 2.32), and Empowerment (A2, M = 2.30), indicating relatively positive perceptions of social support, intellectual engagement, and empowerment among participants. The lowest mean scores occurred in questions representing Physical Wellness (A3, M = 1.47), Financial Wellness (A2, M = 1.54), Empowerment (A3, M = 1.56), and Resilience (A2, M = 1.56), suggesting these areas may represent challenges or opportunities for targeted support.

### 3.2. Internal Consistency Reliability

Reliability was assessed using McDonald’s omega (ω) based on the final 26-item version of the CWQ. There were 225 valid cases (100%). Cases were processed using listwise deletion procedures. The McDonald’s omega of 0.828 supports the reliability of the scale and suggests that the items demonstrate strong internal consistency. Cronbach’s alpha was also strong (0.845; 95% CI: 0.814, 0.873). Because Cronbach’s alpha and omega are similar, there is evidence the scale’s reliability estimates are stable and not being artificially inflated by item redundancy.

### 3.3. Exploratory Factor Analysis

An exploratory factor analysis (EFA) using principal axis factoring with oblique rotation was used for interpretation of factors from the pilot study of the CWQ (Table 3). Due to the expected correlation of subscales, Promax rotation with Kaiser normalization was used in the EFA. The Kaiser–Meyer–Olkin measure verified sampling adequacy (KMO = 0.820) and Bartlett’s test of sphericity was significant (<0.001), thus justifying the factor analysis. The measures of sampling adequacy (MSA) from the anti-image correlation matrix for all variables of the survey were above 0.50 (range: 0.642–0.892), indicating the data as appropriate for factor analysis. Low off-diagonal partial correlations showed independent residual variance.

Initial communalities were inspected to evaluate item intercorrelations (Table 3). All but three items (Occupational Wellness A2, Physical Wellness A3, Financial Wellness A2) had values above 0.30 indicating sufficient shared variance. Six factors explaining 56.44% of variance were retained based on Eigenvalues > 1 and visual inspection of a scree plot (Table 4). The factor correlation matrix shows low to moderate relationships with two having moderately high correlation (0.516), justifying the use of oblique rotation (Table 5).

The structure matrix was used for interpretation due to the expectation of correlated factors and identifies cross-loadings with results > 0.40 considered significant (Table 6). Examination of the structure matrix revealed a six-factor solution. Factor 1 items represent a broad Connected (Social-Spiritual) Well-Being (0.364–0.764), with strongest loadings from spiritual and social well-being items. Factor 2 items reflect Personal Agency (Empowerment and Intellectual) (0.444–0.733), with empowerment and intellectual wellness items loading most strongly. Factor 3 items capture Empowerment and Adaptive Capacity (0.390–0.693). Factor 4 items represent Functional Capacity and Stability (0.452–0.772). Factor 5 items reflect Resilience and Self-Regulation (0.512–0.861), while Factor 6 items represent Occupational Wellness (0.537–0.684).

Although a strong loading was found for Occupational Wellness A3 with Factor 6 (0.684), cross-loading is noted for Factor 1 (0.470). Weak discriminant validity due to cross-loadings are noted with Resilience A1 (Factor 1: 0.495, Factor 2: 0.501, Factor 5: 0.513); Financial Wellness A1 (Factor 1: 0.512, Factor 6: 0.537); Intellectual Wellness A3 (Factor 1: 0.451, Factor 5: 0.512); and Psychological Wellness A1 (Factor 1: 0.436, Factor 2: 0.465). The corresponding pattern matrix is presented in Table 7.

The emergence of a six-factor solution does not indicate a reduction in the number of wellness dimensions comprising the CWU. Instead, the factor analytic results identified broader latent constructs that account for the relationships among questionnaire items while retaining the theoretical content of the original dimensions. Each of the original wellness dimensions remains conceptually represented within the six-factor framework through observed factor loadings and shared thematic relationships. Thus, the revised structure reflects an empirically derived reorganization of wellness domains for measurement purposes rather than a narrowing of the conceptual model of caregiver wellness.

### 3.4. Correlation Analysis of Subscales

The strongest relationship was observed between Spiritual Wellness and Social Wellness (*r* = 0.623, *p* < 0.001), indicating substantial shared variance between these dimensions (Table 8). A second strong association emerged between Empowerment and Resilience (*r* = 0.532, *p* < 0.001), suggesting that caregivers who perceive greater personal agency also tend to report higher resilience. Most remaining correlations fell within the moderate range (*r* = 0.301–0.470), supporting the theoretical expectation that the wellness dimensions are related but distinct constructs. This pattern of moderate-to-strong intercorrelations provides evidence of convergent validity, while the absence of excessively high correlations (e.g., >0.80) suggests that the subscales retain adequate discriminant validity and are not measuring identical aspects of caregiver wellness. The discussion of correlations among the CWQ subscales were noted as convergent or discriminant validity.

## 4. Discussion

The CWQ was originally hypothesized to include nine subscales corresponding to the themes that encompass all nine constructs in the CWU, each contributing uniquely to overall well-being. The retained items continued to represent these theoretical dimensions but were organized into six broader, empirically derived factors, reflecting the multidimensional nature of caregiver wellness (Table 9). Prior to psychometric testing, one survey item was removed because corresponding data were not captured in the dataset.

Following completion of the factor analysis, the questionnaire items were mapped to their corresponding six subscales. The researchers independently reviewed the items and identified themes associated with each newly derived factor. Based on the content and grouping of the items, the researchers used Microsoft Copilot [17] to generate potential titles for the constructs. They subsequently compared their independent findings, discussed the proposed labels, and reached consensus on the final titles. The resulting model demonstrates that these dimensions are interconnected and combine into six broader wellness constructs.

The first subscale, Connected Well-Being (Table 10), captures the degree to which caregivers experience meaningful relationships, emotional well-being, social belonging, a sense of purpose, and spiritual connectedness. Drawing on the original CWU dimensions of social, spiritual, psychological, physical, and occupational wellness, this subscale captures the relational and meaning-centered aspects of wellness that contribute to a caregiver’s sense of connection with self, others, and broader sources of meaning and purpose. It highlights the role of emotional support, social engagement, and spiritual resources in sustaining resilience and overall well-being for caregivers.

The second subscale, Personal Agency, centers on a caregiver’s capacity for self-determination, informed decision-making, personal growth, and effective management of life circumstances. Integrating elements of empowerment, intellectual wellness, psychological and physical wellness, it emphasizes self-efficacy, autonomy, knowledge, and personal initiative. Personal Agency represents an individual’s perceived ability to influence outcomes through informed action and confidence, fostering proactive engagement with caregiving responsibilities and challenges.

The third subscale, Empowered Adaptive Capacity, reflects a caregiver’s ability to adapt effectively to caregiving demands, recover from adversity, derive meaning from challenging experiences, and leverage personal strengths to support continued growth and resilience. This factor integrates elements of empowerment, resilience, and spiritual, intellectual as well as psychological wellness. Its core emphasis is on adaptation, meaning-making, and personal development. Empowered Adaptive Capacity encompasses the cognitive, spiritual, and psychological resources that enable caregivers to respond constructively to stressors, maintain a sense of purpose, and sustain an empowered approach to caregiving.

The fourth subscale, Functional Capacity, describes a caregiver’s ability to maintain daily functioning, physical health, financial stability, and effective caregiving performance. This factor integrates the CWU dimensions of financial, physical, and occupational well-being, representing the practical resources and capacities that support both caregiving responsibilities and personal independence. Functional Capacity encompasses the tangible foundations of wellness necessary for sustaining caregiver effectiveness and overall well-being.

The fifth subscale, Resilient Self-Regulation, concerns a caregiver’s capacity to manage stress, regulate emotions and behaviors, exercise self-control, and maintain well-being through adaptive coping strategies. This factor integrates elements of resilience and intellectual wellness, emphasizing stress management, behavioral adaptation, and effective coping. Resilient Self-Regulation represents the self-management processes that enable caregivers to navigate ongoing demands, maintain psychological stability, and sustain effective functioning over time.

The sixth subscale, Occupational Wellness, addresses the extent to which caregivers experience meaningful work, productive engagement, role fulfillment, financial security, and balance across caregiving, employment, and other life responsibilities. This factor highlights the importance of occupational and financial resources in supporting caregiver well-being and sustaining long-term engagement in both caregiving and workforce roles.

Although items on a psychosocial questionnaire may be expected to exhibit cross-loadings, certain items with weak discriminant validity will be evaluated for revision to better capture the primary factor each item is intended to represent. These results underscore the importance of ongoing item evaluation to ensure comprehensive and conceptually balanced representation of caregiver wellness constructs.

The overlap among factors is consistent with multidimensional theories of well-being, which conceptualize wellness as an integrated system of interrelated domains rather than a set of discrete constructs [18,19]. For example, Ryff and Keyes [18] demonstrated that dimensions such as positive relationships, purpose in life, personal growth, autonomy, self-acceptance, and environmental mastery are distinct yet interconnected components of a higher-order well-being construct. Similarly, Ryff [18] emphasized that well-being emerges through the dynamic interaction of psychological, social, and meaning-centered dimensions of human functioning. Consistent with these theoretical perspectives, the overlap observed among the CWQ subscales suggests that caregiver wellness is best understood as a holistic construct characterized by the reciprocal influence of relational, psychological, physical, spiritual, and occupational resources. Together, these findings support the premise that caregiver well-being is sustained through the interaction of multiple, mutually reinforcing dimensions rather than any single domain in isolation. However, these conclusions should be interpreted cautiously and limited to preliminary evidence supporting the instrument’s overall internal consistency and a provisional six-factor exploratory structure. Further psychometric investigation is necessary to establish factor-level reliability, convergent and criterion-related validity, test–retest reliability, and independent confirmation of the factor structure through confirmatory factor analysis.

### 4.1. Limitations

As this study represents the first large-scale psychometric evaluation of the CWQ, the findings should be considered preliminary and foundational rather than conclusive. One item was excluded because it was not adequately represented within the available dataset. The use of a convenience sample limits the external validity of the findings. Because participants were recruited through a single nonprofit caregiver support organization and self-selected to complete the questionnaire, the sample may not fully represent the broader caregiver population. Future evaluation of the CWQ’s psychometric properties in larger, more geographically, culturally, and occupationally diverse samples is therefore needed to establish the generalizability of the six-subscale structure.

### 4.2. Implications for Future Research

Future research should employ confirmatory factor analysis (CFA) to evaluate the fit of the emergent six-factor model. Additional refinement efforts should include the development and testing of new candidate items, assessment of content coverage within each wellness domain, and examination of item wording, cross-loadings, and factor stability. Studies should also investigate subscale reliability; measurement invariance across caregiver populations, including family caregivers and frontline direct caregivers (healthcare workers); and criterion-related validity using established measures of caregiver burden, stress, resilience, and quality of life. Expanding response options and evaluating measurement equivalence across diverse caregiver groups may enhance the instrument’s sensitivity and applicability. Collectively, these investigations would strengthen the psychometric evidence supporting the CWQ as a valid, reliable, and practically useful measure of caregiver wellness for both research and applied settings.

## 5. Conclusions

The secondary analysis provides early evidence that the CWQ is more than an informational self-help tool; it has potential as a structured wellness measure.

The findings support continued psychometric evaluation, including confirmatory factor analysis and further domain-level reliability testing. The results also clarify how the tool can identify patterns of caregiver need while preserving its original empowerment-centered purpose.

The revised CWU demonstrates that caregiver wellness extends beyond the presence of individual wellness domains and instead emerges from the dynamic interaction of relational connectedness, personal empowerment, adaptive resilience, functional resources, self-regulatory processes, and occupational fulfillment. Conceptualizing caregiver wellness as a second-order construct provides a stronger theoretical foundation for assessment and intervention development, allowing wellness support to target broader, integrated patterns of functioning rather than isolated dimensions. However, the six-factor structure identified in this exploratory analysis should be considered preliminary and interpreted with appropriate caution. Additional psychometric evaluation, including confirmatory factor analysis in independent and more diverse caregiver samples, is needed to verify the stability, replicability, and overall fit of the proposed model. Nevertheless, this integrative six-factor framework offers a promising holistic approach for understanding and promoting sustainable caregiver well-being across diverse caregiving contexts.

## Figures and Tables

**Table 1:** Caregiver Wellness Questionnaire (CWQ) Subscales and Interpretation Across Nine Wellness Dimensions.

Wellness Dimension	Interpretation
Occupational Wellness	Lower scores indicate occupational wellness strengths; higher scores indicate areas for improvement.
Physical Wellness	Lower scores indicate physical wellness strengths; higher scores indicate areas for improvement.
Psychological Wellness	Lower scores indicate psychological wellness strengths; higher scores indicate areas for improvement.
Intellectual Wellness	Lower scores indicate intellectual wellness strengths; higher scores indicate areas for improvement.
Social Wellness	Lower scores indicate social wellness strengths; higher scores indicate areas for improvement.
Resilience	Lower scores indicate resilience strengths; higher scores indicate areas for improvement.
Empowerment	Lower scores indicate empowerment strengths; higher scores indicate areas for improvement.
Spiritual Wellness	Lower scores indicate spiritual wellness strengths; higher scores indicate areas for improvement.
Financial Wellness	Lower scores indicate financial wellness strengths; higher scores indicate areas for improvement.

Note: Each scale includes 3 items. Score range for each scale is 3–9. Score Interpretation Guide; 3–4: Wellness Strength; 5–6: Moderate Attention Needed; 7–9: Priority Area for Improvement.

**Table 2:** Descriptive Statistics (*N* = 225).

Variable	Mean	SD
Occupational Wellness—A1	1.78	0.83
Occupational Wellness—A2	1.84	0.81
Occupational Wellness—A3	1.80	0.63
Physical Wellness—A1	2.12	0.80
Physical Wellness—A2	1.74	0.72
Physical Wellness—A3	1.47	0.71
Psychological Wellness—A1	1.75	0.71
Psychological Wellness—A2	2.10	0.75
Psychological Wellness—A3	1.89	0.70
Intellectual Wellness—A1	1.68	0.72
Intellectual Wellness—A2	2.32	0.73
Intellectual Wellness—A3	1.60	0.66
Social Well-being—A1	2.16	0.77
Social Well-being—A2	2.33	0.75
Spiritual Well-being—A1	2.02	0.80
Spiritual Well-being—A2	2.13	0.75
Spiritual Well-being—A3	1.88	0.75
Empowerment—A1	1.74	0.73
Empowerment—A2	2.30	0.74
Empowerment—A3	1.56	0.50
Resilience—A1	2.00	0.66
Resilience—A2	1.56	0.70
Resilience—A3	2.17	0.72
Financial Wellness—A1	1.98	0.83
Financial Wellness—A2	1.54	0.76
Financial Wellness—A3	1.96	0.84

Note: Descriptive statistics for the 26 items retained in the Caregiver Wellness Questionnaire across nine wellness dimensions, based on responses from 225 caregivers. All items were measured on a 3-point response scale, with observed scores ranging from 1 to 3, except for Empowerment Item A3, which had a maximum observed score of 2.

**Table 3:** Communalities.

Subscales	Initial
Occupational Wellness—A1	0.308
Occupational Wellness—A2	0.271
Occupational Wellness—A3	0.481
Physical Wellness—A1	0.323
Physical Wellness—A2	0.350
Physical Wellness—A3	0.240
Psychological Wellness—A1	0.388
Psychological Wellness—A2	0.327
Psychological Wellness—A3	0.446
Intellectual Wellness—A1	0.438
Intellectual Wellness—A2	0.325
Intellectual Wellness—A3	0.392
Social Well-being—A1	0.524
Social Well-being—A2	0.564
Spiritual Well-being—A1	0.476
Spiritual Well-being—A2	0.617
Spiritual Well-being—A3	0.411
Empowerment—A1	0.501
Empowerment—A2	0.387
Empowerment—A3	0.308
Resilience—A1	0.503
Resilience—A2	0.458
Resilience—A3	0.401
Financial Wellness—A1	0.444
Financial Wellness—A2	0.293
Financial Wellness—A3	0.401

Extraction Method: Principal Axis Factoring.

**Table 4:** Total Variance Explained.

Factor	Initial Eigenvalues			Rotation Sums of Squared Loadings ^a^
	Total	% of Variance	Cumulative %	Total
1	5.889	22.651	22.651	4.532
2	2.833	10.898	33.549	3.257
3	2.226	8.562	42.111	2.284
4	1.454	5.591	47.702	1.908
5	1.202	4.624	52.326	2.448
6	1.070	4.114	56.440	1.789
7	0.966	3.715	60.155	
8	0.925	3.559	63.714	
9	0.877	3.374	67.088	
10	0.842	3.238	70.326	
11	0.763	2.935	73.261	
12	0.729	2.805	76.066	
13	0.669	2.575	78.641	
14	0.621	2.387	81.028	
15	0.592	2.277	83.305	
16	0.564	2.170	85.475	
17	0.500	1.923	87.398	
18	0.472	1.817	89.215	
19	0.445	1.713	90.928	
20	0.430	1.654	92.582	
21	0.396	1.523	94.105	
22	0.377	1.451	95.556	
23	0.329	1.265	96.820	
24	0.308	1.184	98.004	
25	0.275	1.059	99.063	
26	0.244	.937	100.000	

Extraction Method: Principal Axis Factoring. ^a^ When factors are correlated, sums of squared loadings cannot be added to obtain a total variance.

**Table 5:** Factor Correlation Matrix.

Factor	Correlation Matrix					
	1	2	3	4	5	6
1	1.000	0.393	0.210	0.157	0.311	0.243
2	0.393	1.000	−0.020	0.054	0.516	0.278
3	0.210	−0.020	1.000	0.364	0.041	−0.075
4	0.157	0.054	0.364	1.000	0.285	0.005
5	0.311	0.516	0.041	0.285	1.000	0.066
6	0.243	0.278	−0.075	0.005	0.066	1.000

Extraction Method: Principal Axis Factoring. Rotation Method: Promax with Kaiser Normalization.

**Table 6:** Structure Matrix from Exploratory Factor Analysis (Principal Axis Factoring with Promax Rotation with Kaiser Normalization).

Variable	Factor 1	Factor 2	Factor 3	Factor 4	Factor 5	Factor 6
Spiritual Well-being—A2	0.764	0.330	0.051	0.066	0.211	0.398
Social Well-being—A2	0.763	0.199	0.367	0.104	0.188	−0.008
Social Well-being—A1	0.719	0.239	0.189	0.093	0.171	0.198
Spiritual Well-being—A1	0.655	0.304	0.034	−0.034	0.185	0.251
Psychological Wellness—A3	0.592	0.042	−0.028	0.098	0.349	0.178
Physical Wellness—A1	0.516	0.156	0.134	0.205	0.060	0.244
Occupational Wellness—A1	0.364	0.146	0.115	0.110	0.174	0.358
Empowerment—A1	0.317	0.733	0.082	0.010	0.475	0.008
Intellectual Wellness—A1	0.296	0.682	−0.029	0.199	0.342	0.179
Empowerment—A3	0.179	0.549	−0.004	0.079	0.310	0.160
Psychological Wellness—A1	0.436	0.465	0.011	0.025	0.326	0.279
Physical Wellness—A3	0.120	0.444	0.128	−0.011	0.108	0.243
Resilience—A3	0.192	0.055	0.693	0.175	−0.064	0.168
Empowerment—A2	0.152	0.081	0.672	0.262	0.127	−0.096
Spiritual Well-being—A3	0.063	−0.012	0.561	0.343	0.182	−0.290
Intellectual Wellness—A2	0.346	−0.091	0.492	0.287	0.012	−0.024
Psychological Wellness—A2	0.276	0.064	0.390	0.204	−0.086	0.142
Financial Wellness—A3	0.137	−0.034	0.317	0.772	0.112	−0.100
Financial Wellness—A2	0.058	0.213	0.148	0.488	0.209	0.167
Occupational Wellness—A2	0.154	0.047	0.320	0.463	0.204	0.150
Physical Wellness—A2	−0.077	−0.005	0.410	0.452	0.217	−0.092
Resilience—A2	0.222	0.461	0.020	0.209	0.861	0.158
Resilience—A1	0.495	0.501	0.082	0.152	0.513	0.293
Intellectual Wellness—A3	0.451	0.387	0.057	0.264	0.512	0.191
Occupational Wellness—A3	0.470	0.334	−0.042	0.202	0.313	0.684
Financial Wellness—A1	0.512	0.328	0.071	−0.030	0.147	0.537

Note: Table entries represent structure coefficients (factor loadings) obtained from principal axis factoring with Promax rotation with Kaiser Normalization. Higher absolute values indicate stronger associations between items and factors.

**Table 7:** Pattern Matrix.

Subscales	Factor					
	1	2	3	4	5	6
Social Well-being—A2	0.795	−0.057	0.222	−0.098	0.000	−0.168
Spiritual Well-being—A2	0.742	−0.010	−0.082	−0.014	−0.022	0.215
Social Well-being—A1	0.727	−0.038	0.062	−0.033	−0.031	0.039
Spiritual Well-being—A1	0.654	0.034	−0.053	−0.117	−0.006	0.080
Physical Wellness—A1	0.514	−0.015	−0.022	0.175	−0.150	0.131
Psychological Wellness—A3	0.431	0.370	−0.134	0.055	0.016	−0.041
Psychological Wellness—A1	0.278	0.266	−0.015	−0.060	0.111	0.129
Empowerment—A1	0.039	0.710	0.108	−0.113	0.138	−0.199
Intellectual Wellness—A1	0.041	0.708	−0.101	0.217	−0.092	−0.030
Empowerment—A3	−0.058	0.548	0.001	0.050	0.030	0.020
Physical Wellness—A3	−0.114	0.519	0.200	−0.059	−0.126	0.150
Resilience—A3	0.006	0.082	0.737	−0.062	−0.134	0.208
Empowerment—A2	−0.029	0.091	0.673	−0.002	0.067	−0.068
Spiritual Well-being—A3	−0.049	−0.001	0.500	0.125	0.158	−0.251
Intellectual Wellness—A2	0.345	−0.199	0.372	0.119	−0.040	−0.023
Physical Wellness—A2	−0.249	−0.017	0.339	0.311	0.202	−0.016
Psychological Wellness—A2	0.209	0.079	0.322	0.120	−0.246	0.109
Financial Wellness—A3	0.082	−0.014	0.006	0.794	−0.126	−0.111
Financial Wellness—A2	−0.134	0.201	0.010	0.495	−0.004	0.143
Occupational Wellness—A2	0.010	−0.085	0.193	0.360	0.123	0.176
Resilience—A2	−0.084	0.005	0.026	−0.041	0.887	0.120
Intellectual Wellness—A3	0.291	0.065	−0.062	0.138	0.347	0.074
Resilience—A1	0.280	0.183	0.027	−0.003	0.321	0.154
Occupational Wellness—A3	0.289	−0.045	−0.121	0.152	0.168	0.606
Financial Wellness—A1	0.387	0.067	0.067	−0.119	−0.005	0.431
Occupational Wellness—A1	0.277	−0.110	0.069	0.013	0.117	0.318

Extraction Method: Principal Axis Factoring. Rotation Method: Promax with Kaiser Normalization. Rotation converged in 9 iterations.

**Table 8:** Correlation Analysis of Caregiver Wellness Questionnaire (CWQ) Subscales.

Subscales	Occ Well	Phys Well	Psych Well	Inte Well	Soc WB	Spirit WB	Empower	Resil	Fin Well
Occ Well	1	0.354 **	0.308 **	0.341 **	0.344 **	0.384 **	0.193 **	0.388 **	0.436 **
Phys Well	0.354 **	1	0.299 **	0.438 **	0.287 **	0.264 **	0.301 **	0.341 **	0.345 **
Psych Well	0.308 **	0.299 **	1	0.461 **	0.470 **	0.459 **	0.381 **	0.384 **	0.348 **
Inte Well	0.341 **	0.438 **	0.461 **	1	0.432 **	0.426 **	0.429 **	0.450 **	0.412 **
Soc WB	0.344 **	0.287 **	0.470 **	0.432 **	1	0.623 **	0.257 **	0.383 **	0.261 **
Spirit WB	0.384 **	0.264 **	0.459 **	0.426 **	0.623 **	1	0.357 **	0.410 **	0.363 **
Empower	0.193 **	0.301 **	0.381 **	0.429 **	0.257 **	0.357 **	1	0.532 **	0.241 **
Resil	0.388 **	0.341 **	0.384 **	0.450 **	0.383 **	0.410 **	0.532 **	1	0.346 **
Fin Well	0.436 **	0.345 **	0.348 **	0.412 **	0.261 **	0.363 **	0.241 **	0.346 **	1

Note: Occ Well, Occupational Wellness. Phys Well, Physical Wellness. Psych Well, Psychological Wellness. Inte Wel, Intellectual Wellness. Soc WB, Social Well-being. Spirit WB, Spiritual Well-being. Empower, Empowerment. Resil, Resilience. Fin Well, Financial Well-being. Values are Pearson product-moment correlations. Only statistically significant correlations (*p* < 0.001) are reported. Correlations of 0.30–0.49 were interpreted as moderate, and correlations ≥ 0.50 were interpreted as strong. Subscales (Moderate to Strong Correlation) ** *p* < 0.001.

**Table 9:** The Caregiver Wellness Six-Factor Model: A Second-Order Model Integrating the Nine U-Model Wellness Dimensions.

Factor	Definition	Primary CWU Dimensions Represented	Core Contribution
**Connected Well-Being**	Reflects the extent to which caregivers experience meaningful relationships, emotional well-being, social belonging, purpose, and spiritual connectedness.	Social Wellness; Spiritual Wellness; Psychological Wellness	Connection, belonging, purpose, and emotional well-being
**Personal Agency**	Reflects a caregiver’s perceived capacity to direct life circumstances, make informed decisions, exercise self-determination, pursue learning, and influence personal outcomes.	Empowerment; Intellectual Wellness; Psychological Wellness	Self-efficacy, growth, learning, and informed choice
**Empowered Adaptive Capacity**	Refers to the ability to adapt effectively to caregiving demands, recover from adversity, derive meaning from challenges, and apply personal strengths to caregiving situations.	Resilience; Empowerment; Spiritual Wellness; Intellectual Wellness	Adaptation, recovery, meaning-making, and personal growth
**Functional Capacity**	Reflects the practical resources, supports, and abilities necessary to maintain daily functioning and sustain caregiving responsibilities.	Physical Wellness; Financial Wellness; Occupational Wellness	Resources, health, financial security, and daily functioning
**Resilient Self-Regulation**	Describes the capacity to manage stress, regulate emotions and behaviors, maintain self-control, and sustain well-being through adaptive coping processes.	Resilience; Empowerment; Intellectual Wellness	Self-control, stress management, and adaptive coping
**Occupational Wellness**	Reflects the degree to which caregivers experience meaningful work, productive engagement, role satisfaction, financial stability, and balance between caregiving and other responsibilities.	Occupational Wellness; Financial Wellness	Meaningful work, role fulfillment, and financial stability

Note: The six-factor structure emerged from exploratory factor analysis of the CWQ. The model conceptualizes caregiver wellness as a second-order construct comprising Connected Well-Being, Personal Agency, Empowered Adaptive Capacity, Functional Capacity, Resilient Self-Regulation, and Occupational Wellness. Original U-Model wellness dimensions are represented within each factor based on observed factor loadings and conceptual relationships.

**Table 10:** Emergent Factors, Higher-Order Themes, and Original U-Model Components.

Factor	Primary Theme	Constituent U-Model™ Dimensions
Connected Well-Being	Connection, belonging, and purpose	Social Wellness; Spiritual Wellness; Psychological Wellness; Physical Wellness; Occupational Wellness
Personal Agency	Self-determination and growth	Empowerment; Intellectual Wellness; Psychological Wellness; Physical Wellness
Empowered Adaptive Capacity	Adaptation and resilience	Resilience; Empowerment; Spiritual Wellness; Intellectual Wellness; Psychological Wellness
Functional Capacity	Daily functioning and resources	Financial Wellness; Occupational Wellness; Physical Wellness
Resilient Self-Regulation	Adaptive coping and self-management	Resilience; Intellectual Wellness
Occupational Wellness	Meaningful work and role fulfillment	Occupational Wellness; Financial Wellness

Note: The table presents the six-factor structure derived from exploratory factor analysis and its correspondence to higher-order themes and original CWU components. The right-hand column identifies the contributing wellness dimensions from the original nine-domain CWU. The figure is intended as a conceptual model illustrating the theoretical organization of caregiver wellness constructs following factor refinement.

## Data Availability

The data are proprietary data, but they are available for peer review purposes. The raw data supporting the conclusions of this article will be made available by the authors on request.

## Abbreviations

The following abbreviations are used in this manuscript:

CWDC	The Caregiver Wellness Domain Compendium
EFA	Exploratory Factor Analysis
CWU	Caregiver Wellness: U-Model
CWQ	Caregiver Wellness: U-Model Questionnaire
CSS	Caregiver Support Services
